# Lymph node ratio is a better prognosticator than lymph node status for gastric cancer: A retrospective study of 138 cases

**DOI:** 10.3892/ol.2013.1615

**Published:** 2013-10-10

**Authors:** WEI-JUAN ZENG, WEN-QIN HU, LIN-WEI WANG, SHU-GUANG YAN, JIAN-DING LI, HAO-LIANG ZHAO, CHUN-WEI PENG, GUI-FANG YANG, YAN LI

**Affiliations:** 1 Departments of Oncology and Pathology, Zhongnan Hospital of Wuhan University, Hubei Key Laboratory of Tumor Biological Behaviors and Hubei Cancer Clinical Study Center, Wuhan, Hubei 430071, P.R. China; 2 Department of Surgery, Heji Hospital Affiliated to Changzhi Medical College, Changzhi, Shanxi 046000, P.R. China; 3 Department of Medical Imaging, The First Affiliated Hospital of Shanxi Medical University, Taiyuan, Shanxi 030001, P.R. China; 4 Department of General Surgery, Shanxi University Hospital, Taiyuan, Shanxi 046000, P.R. China

**Keywords:** gastric cancer, lymph node ratio, prognosis

## Abstract

To study the clinical significance of lymph node ratio (LNR) in gastric cancer (GC), this study analyzed 613 patients with GC who underwent surgical resection. Of 613 patients with GC, 138 patients who had >15 lymph nodes (LNs) resected and radical resection were enrolled into the final study. All major clinicopathological data were entered into a central database. LNR was defined as the ratio of the number of metastatic LNs to the number of removed LNs. In order to determine the best cut-off points for LNR, the log-rank test and X-tile were used. LNR was then substituted for lymph node status (pN) in the 7th American Joint Committee on Cancer tumor-node-metastases (TNM) staging system and this was defined as the tumor-node ratio-metastases (TRM) staging system. Pearson's correlation coefficient (r) was used to study the correlations among the number of removed LNs, pN and LNR. The Kaplan-Meier survival curve was used to study the survival status, and the log-rank test and Cox proportional hazards model were used to identify the independent factors for survival. Receiver operating characteristic curve analysis was used to determine the predictive value of the parameters. By the time of last follow-up (median follow-up period, 38.3 months; range, 9.9–97.7 months), the median overall survival (OS) was 23.9 months [95% confidence interval (CI), 18.8–29.0 months]. The 1-, 2-, 3- and 5-year survival rates were 76.8, 57.2, 50.0 and 46.4%, respectively. The cut-off points were 0, 0.5 and 0.8 (R0, LNR=0; R1, LNR ≤0.5; R2, 0.5> LNR ≤0.8; and R3, LNR >0.8). Univariate and multivariate analyses revealed that both LNR and pN were independent prognostic factors for GC. LNR could better differentiate OS in patients than LN. In addition, the TRM staging system was better at predicting the clinical outcomes than the TNM staging system, and LNR was better than pN. In conclusion, LNR was a better prognosticator than pN for GC.

## Introduction

Gastric cancer (GC) is the fourth most common malignancy with ~1 million patients diagnosed with GC worldwide per year, and the second leading cause of cancer-related mortality worldwide with 800,000 fatalities per year (1), though the prevalence and mortality of GC have gradually decreased (2). In China, ~0.4 million new cases of GC and 0.3 million fatalities occurred each year, making it the third leading cause of cancer-related mortality (3). In addition, the outcome of GC remains poor with a 5-year survival rate of only ~20–25% (4).

Accurate prediction of the prognosis of patients with GC is crucial, as surgery is the most important therapeutic approach (5). It helps to define which patients with GC should receive secondary treatments, such as chemotherapy and/or radiotherapy, which are largely dependent on clinical staging (6,7).

The prognosis of GC is closely related to the tumor stage, including the depth of tumor invasion, lymph node status and distant metastases (8–10). The most commonly used staging system of GC is proposed by the American Joint Committee on Cancer (AJCC) and is known as the AJCC tumor-node-metastases (TNM) staging system. In 2010, the 7th edition of the AJCC gastric cancer staging manual was ascertained, resulting in much controversy (11). Certain studies confirmed that the 7th AJCC TNM staging system was superior to the 6th AJCC TNM staging system (12–14), while other studies confirmed the 6th was better for prognostic stratification (15,16).

In recent years, lymph node ratio (LNR), defined as the ratio of the number of metastatic lymph nodes (LNs) to the number of removed LNs, has gained increasing attention in researches because of its lymph node status (pN) in AJCC TNM staging system (17–19). However, the analytical methods of these studies were commonly the same and no in depth investigations have been conducted.

In this study, GC patients with radical resection and extended lymphatic resection were selected, as R0 resection with D2 lymphadenectomy is regarded as the standard surgical technique in Eastern Asian countries (7). Comprehensive analytical methods were used to evaluate whether LNR was a superior prognosticator compared with pN for GC.

## Patients and methods

### Patients

This retrospective study initially consisted of 613 patients with GC who underwent resection from three tertiary referral hospitals from January 2004 to August 2011. All the clinicopathological information was available, including demographic variables, underlying co-morbidities, surgical modality, lab and image study information, pathological reports, pre- and postoperative therapies, and follow-up information. Among these patients with GC, only those who had >15 LNs resected and radical resection were enrolled into the final study. Patients who had palliative resection, ≤15 LNs resected and incomplete follow-up information were excluded, as this method was more suitable for those patients with >15 LNs resected. In total, 138 patients were enrolled into the final study.

The patients were followed up every 3 months during the first 2 years after surgery, every 6 months during the third postoperative year and every year thereafter. All the follow-up information was entered into a database.

### Tumor-node-ratio-metastases (TRM) staging system

For defining the TRM staging system, two recognized methods were used to determine the best cut-off points for LNR. One was the commonly used cut-off approach using the log-rank test, the other was X-tile as reported by Wang *et al*(17). X-tile determines the optimal cut-off points of LNR by taking LNR as a continuous variable. Compared with the commonly used cut-off approach, X-tile controls for the inflated type I error problem and minimizes information loss. LNR was then substituted for pN in the 7th AJCC TNM staging system to generate the TRM staging system as the N classification of the 7th AJCC TNM staging system is thought to be superior (13).

### Statistical analysis

All data were analyzed using SPSS 17.0 statistical software package (SPSS, Inc., Chicago, IL, USA). Pearson's correlation coefficient (r) was used to study the correlations among the number of removed LNs, pN and LNR. The Kaplan-Meier survival curve was used to study the survival status, and the log-rank test and Cox proportional hazards model were used to identify the independent factors for survival. Receiver operating characteristic (ROC) curve analysis was used to determine the predictive value of the parameters. Two-sided P<0.05 was considered to indicate a statistically significant difference.

## Results

### Characteristics of patients

Among 613 GC patients who had undergone resection from three tertiary referral hospitals between January 2004 and August 2011, 138 were enrolled into the final study. By the time of the last follow-up (May 31, 2012), 76 mortalities had occurred. The median number of removed LNs was 21 (range 16–47). The median age of patients was 56 years (range 27–79 years), and the male-to-female ratio was 2.54 to 1. Detailed information is listed in Table I.

### TRM staging system

**Figure 1:**
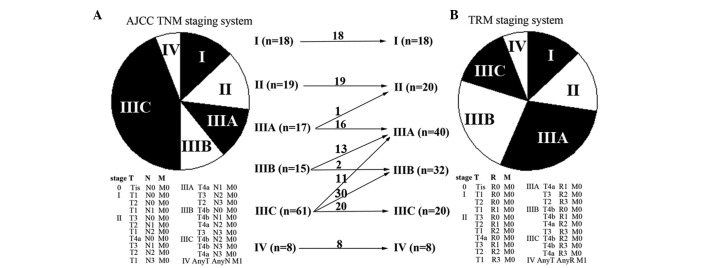
Patient distribution in the 7th American Joint Committee on Cancer (AJCC) (A) tumor-node-metastases (TNM) staging system and (B) tumor-node-ratio-metastases (TRM) staging system. Compared with the 7th AJCC TNM staging system, 55 (39.9%) GC patients were downstaged and no patients were upstaged in the TRM staging system.

According to the commonly used cut-off approach by the log-rank test, three cut-off points were generated: 0, 0.50 and 0.80. According to X-tile (http://www.tissuearray.org/rimmlab/), patients with LNR=0 were fixed into one group, as it has been demonstrated that their prognosis was significantly different from patients with LNR >0 (20). The remaining patients were analyzed and the two other cut-off points were 0.48 and 0.79. Considering the log-rank test results and clinical feasibility, the final cut-off points for LNR were set as 0, 0.5 and 0.8. Four subgroups were then determined (R0, LNR=0; R1, LNR ≤0.5; R2, 0.5> LNR ≤0.8; and R3, LNR >0.8), and the TRM staging system was generated. Compared with the 7th AJCC TNM staging system, 55 (39.9%) GC patients were downstaged and no patients were upstaged in the TRM staging system (Fig. 1).

### Correlations between the number of removed LNs, pN and LNR

**Figure 2:**
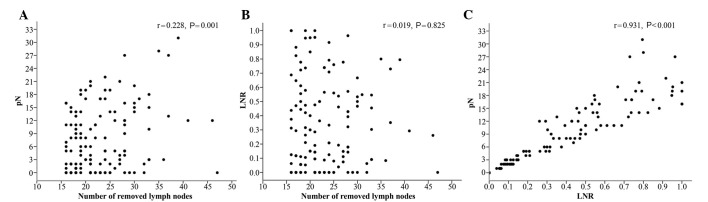
Pearson's correlation tests. (A) Significant correlation between the number of removed lymph nodes (LNs) and lymph node status (pN). (B) Non-significant correlation between the number of removed LNs and lymph node ratio (LNR). (C) Significant correlation between pN and LNR.

There was a significant correlation between the number of removed LNs and pN (r=0.228, P=0.001) (Fig. 2A). There was no significant correlation between the number of removed LNs and LNR (r=0.019, P=0.825) (Fig. 2B). The difference between pN and LNR was statistically significant (r=0.931, P<0.001) (Fig. 2C). These results demonstrated that LNR was not influenced by surgery; however, pN was.

### Univariate and multivariate analyses

By the Kaplan-Meier curve and log-rank test, nine factors were identified as possible determinants on overall survival (OS), including cancer site (P=0.020), tumor invasion (P=0.004), pN (P<0.001), LNR (P<0.001), distant metastases (P<0.001), TNM staging (P<0.001), TRM staging (P<0.001), surgery type (P=0.044) and postoperative serious adverse events (SAEs) (P<0.001). All factors were then integrated into multivariate analysis using Cox proportional hazards model, and both LNR and pN were found to be independent prognostic factors (Table II).

### Comparison of the discriminative power between pN and LNR for OS

**Figure 3:**
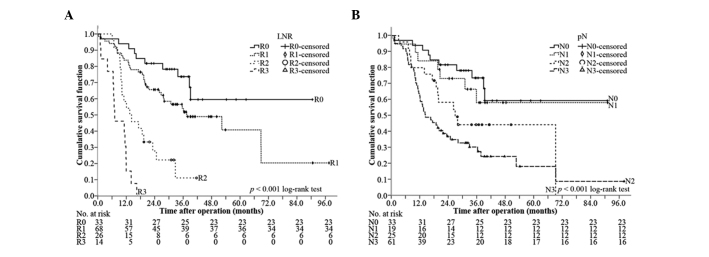
Kaplan-Meier survival curves, classified by (A) lymph node ratio (LNR) and (B) lymph node status (pN). The LNR could better divide the patients into four different groups than pN.

The median OS of R0, R1, R2 and R3 was 64.4 months [95% confidence interval (CI), 50.9–77.9 months], 37.8 months (95% CI, 19.6–56.0 months), 13.8 months (95% CI, 6.4–21.2 months) and 7.5 months (95% CI, 2.2–12.7 months), respectively (P<0.001, overall comparison; P=0.071 for R0 vs. R1; P<0.001 for R1 vs. R2; and P=0.001 for R2 vs. R3) (Fig. 3A). In comparison, the median OS of N0, N1, N2 and N3 was 64.4 months (95% CI, 50.9–77.9 months), 61.5 months (95% CI, 44.4–78.6 months), 27.0 months (95% CI, 15.4–38.6 months) and 14.6 months (95% CI, 8.4–20.8 months), respectively (P<0.001, all overall comparison; P=0.597 for N0 vs. N1; P=0.168 for N1 vs. N2; P=0.122 for N2 vs. N3) (Fig. 3B). Therefore, LNR could better differentiate OS than LN.

### Comparison of the discriminative power between TRM staging and TNM staging for OS

**Figure 4:**
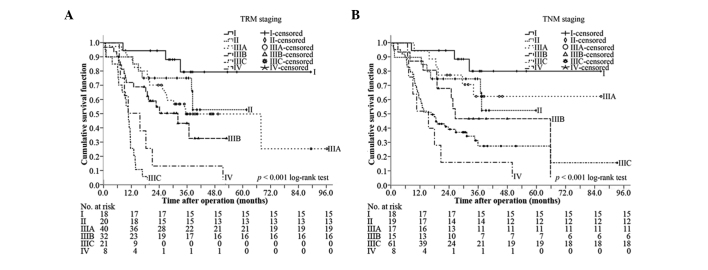
Kaplan-Meier survival curves, classified by (A) tumor-node-ratio-metastases (TRM) staging, and (B) tumor-node-metastases (TNM) staging. The TRM staging could better divide the patients into six different groups than the TNM staging. Censored patients were alive at the time of the most recent follow-up and their survival-time was recorded as the last follow-up date.

At the median follow-up of 38.3 months (range, 9.9–97.7 months), the median OS was 23.9 months (95% CI, 18.8–29.0 months), and the 1-, 2-, 3- and 5-year survival rates were 76.8, 57.2, 50.0 and 46.4%, respectively. Based on the TRM staging, the median OS of stages I, II, IIIA, IIIB, IIIC and IV was 77.2 months (95% CI, 63.4–91.0 months), 44.3 months (95% CI, 33.4–55.1 months), 36.4 months (95% CI, 4.3–68.5 months), 25.0 months (95% CI, 10.1–39.9 months), 11.3 months (95% CI, 9.1–13.5 months) and 11.4 months (95% CI, 2.1–20.7 months), respectively (P<0.001, overall comparison; P=0.228 for I vs. II; P=0.490 for II vs. IIIA; P=0.173 for IIIA vs. IIIB; P<0.001 for IIIB vs. IIIC, P=0.072 for IIIC vs. IV) (Fig. 4A). By comparison, the median OS of stages I, II, IIIA, IIIB, IIIC and IV of TNM staging was 77.2 months (95% CI, 63.4–91.0 months), 43.4 months (95% CI, 32.3–54.5 months), 64.5 months (95% CI, 47.7–81.3 months), 28.0 months (95% CI, 25.0–31.0 months), 14.6 months (95% CI, 8.6–20.5 months) and 11.4 months (95% CI, 2.1–20.7 months), respectively (P<0.001, overall comparison; P=0.190 for I vs. II; P=0.786 for II vs. IIIA; P=0.180 for IIIA vs. IIIB; P=0.181 for IIIB vs. IIIC, P=0.212 for IIIC vs. IV) (Fig. 4B). TRM staging was capable of discriminating stages IIIB and IIIC, but TNM staging could not discriminate any neighboring subgroups.

### Predictive accuracy

**Figure 5:**
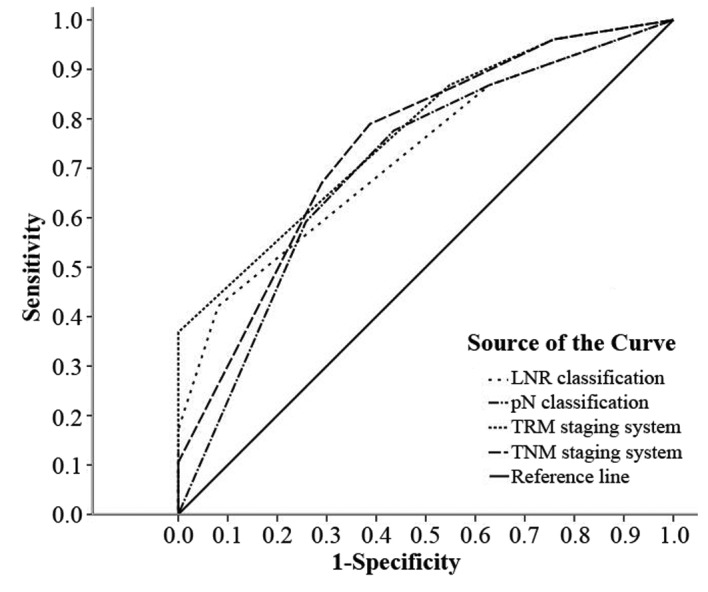
Predictive values of lymph node status (pN) classification, lymph node ratio (LNR), 7th American Joint Committee on Cancer (AJCC) tumor-node-metastases (TNM) staging system and tumor-node-ratio-metastases (TRM) staging system. The TRM staging system could better predict the clinical outcomes compared with the TNM staging system, and LNR was better than pN.

The predictive value of the LNR classification, pN classification, TRM staging system and TNM staging system was further studied by ROC analysis. All of the factors predicted mortality precisely (P<0.01) (Table III). The TRM staging system was better to predict the clinical outcomes than the TNM staging system, and LNR was better than pN (Fig. 5).

## Discussion

Due to the shortcomings of the AJCC TNM staging system, increasing numbers of investigators have shifted their attention to looking for an optimal method. The most popular and the most recognized optimal method was the TRM staging system based on LNR. Table IV lists a number of previous studies on LNR, and these studies confirmed the superiority of the LNR and TRM staging system compared with the AJCC TNM staging system through univariate and multivariate analysis and Kaplan-Meier survival curves (8). In the present study, the cut-off points were 0, 0.50 and 0.80.

In our previous study of GC, patients with stage IIIB and beyond had much poorer OS than other patients. In this study, stage IIIB and beyond accounted for >60% of patients in TNM staging, but <45% in TRM staging, as 24 (17.4%) patients were downstaged to stage IIIA. The prognosis of patients with different classifications could apparently be discriminated, and this may provide a basis for determining secondary treatment.

In routine clinical practice, LN resection in GC patients is generally not up to D2 lymphadenectomy standard, despite D2 lymphadenectomy being regarded as the standard surgical technique in Eastern Asian countries (21,22). Certain studies did not consider this factor in the inclusion criteria (17,23,27,30), weakening the credibility of the results. In this study, only patients with >15 LN resections were included. However, pN is correlated with surgery, whereas LNR is not. Therefore, in univariate and multivariate analyses, both pN and LNR were independent prognostic factors, indicating that LNR was closely associated with prognosis, similar to pN. Moreover, the LNR and TRM staging system could better discriminate subgroups (Figs. 3 and 4), as confirmed by other studies (17,23–27,29). From this perspective, LNR was a better prognosticator than pN.

As to the predictive accuracy analysis, there has been no validated standard. The most commonly used methods were the area under the curve by ROC analysis, the concordance index, explained variation and a summary measure of separation (31). In this study, the TRM staging system had the maximal area under the curve by ROC, and LNR also had a bigger area than pN.

In conclusion, LNR may be a better prognosticator than pN for the following reasons: i) LNR has no correlation with surgery; ii) there were 55 (39.9%) GC patients down-staged and no patients upstaged in the TRM staging system; iii) in univariate and multivariate analysis, both LNR and pN were independent prognostic factors; iv) the LNR and TRM staging system were capable of better differentiating patients than the pN and TNM staging system; v) in ROC analysis, the LNR and TRM staging system have a greater area than the pN and TNM staging system, respectively. The resulting TRM staging system may better predict the clinical outcomes.

## Figures and Tables

**Table I:** The characteristics and univariate analysis of 138 patients with GC.

Variables	n (%)	Events (%)	Median OS (95% CI) (months)	P-value
Hospital				0.374
Zhongnan Hospital	53 (38.4)	34 (64.2)	25.0 (15.4–34.6)	
Heji Hospital	43 (31.2)	21 (48.4)	38.9 (35.0–42.8)	
Hubei Tumor Hospital	42 (30.4)	21 (50.0)	34.1 (27.8–40.4)	
Gender				0.171
Male	99 (71.7)	52 (52.5)	36.4 (25.8–47.0)	
Female	39 (28.3)	24 (61.5)	27.0 (16.3–37.7)	
Age (years)				0.216
≤65	101 (73.2)	52 (51.5)	36.4 (17.5–55.3)	
>65	37 (26.8)	24 (64.9)	25.0 (16.1–33.9)	
Cancer site				0.020
Upper third	31 (22.5)	14 (45.2)	31.1 (25.6–36.5)	
Middle third	27 (19.6)	15 (55.6)	25.0 (10.9–39.1)	
Lower third	70 (50.7)	39 (55.7)	36.4 (25.3–47.5)	
Whole stomach	10 (7.2)	8 (80.0)	8.7 (4.1–13.4)	
Pathological type				0.126
Intestinal	106 (76.8)	57 (53.8)	35.9 (25.7–46.1)	
Diffuse	12 (8.7)	5 (41.7)	25.9 (19.3–32.4)	
Mixed	20 (14.5)	14 (70.0)	14.1 (8.2–20.0)	
Surgery type				0.044
Proximal gastrectomy	36 (26.1)	16 (44.4)	30.7 (25.4–36.0)	
Distant gastrectomy	81 (58.7)	44 (54.3)	36.4 (25.6–47.2)	
Total gastrectomy	21 (15.2)	16 (76.2)	13.4 (1.9–24.9)	
Tumor invasion				0.004
T1	6 (4.3)	2 (33.3)	43.5 (26.3–60.7)	
T2	21 (15.2)	4 (19.0)	75.0 (61.3–88.8)	
T3	1 (0.7)	1 (100.0)	15.8 (15.8–15.8)	
T4a	79 (57.2)	48 (60.8)	28.2 (16.7–39.7)	
T4b	31 (22.5)	21 (67.7)	17.5 (10.4–24.6)	
pN				<0.001
N0	33 (23.9)	10 (30.3)	64.4 (50.9–77.9)	
N1	19 (13.8)	7 (36.8)	61.5 (44.4–78.6)	
N2	25 (18.1)	14 (56.0)	27.0 (15.4–38.6)	
N3	61 (44.2)	45 (73.8)	14.6 (8.4–20.8)	
LNR				<0.001
R0	33 (23.9)	10 (30.3)	64.4 (50.9–77.9)	
R1	68 (49.3)	34 (50.0)	37.8 (19.6–56.0)	
R2	24 (17.4)	19 (79.2)	13.8 (6.4–21.2)	
R3	13 (9.4)	13 (100.0)	7.5 (2.2–12.7)	
Distant metastases				<0.001
M0	128 (92.8)	66 (51.6)	36.4 (27.8–45.0)	
M1	10 (7.2)	10 (100.0)	11.4 (7.6–15.1)	
TNM staging				<0.001
I	18 (13.0)	3 (16.7)	77.2 (63.4–91.0)	
II	19 (13.8)	7 (36.8)	43.4 (32.3–54.5)	
IIIA	17 (12.3)	6 (35.3)	64.5 (47.7–81.3)	
IIIB	15 (10.9)	9 (60.0)	28.0 (25.0–31.0)	
IIIC	61 (44.2)	43 (70.5)	14.6 (8.6–20.5)	
IV	8 (5.8)	8 (100.0)	11.4 (2.1–20.7)	
TRM staging				<0.001
I	18 (13.0)	3 (16.7)	77.2 (63.4–91.0)	
II	20 (14.5)	7 (35.0)	44.3 (33.4–55.1)	
IIIA	40 (29.0)	21 (52.5)	36.4 (4.3–68.5)	
IIIB	32 (23.2)	17 (53.1)	25.0 (10.1–39.9)	
IIIC	20 (14.5)	20 (100.0)	11.3 (9.1–13.5)	
IV	8 (5.8)	8 (100.0)	11.4 (2.1–20.7)	
Postoperative SAE				<0.001
No	118 (85.5)	57 (48.3)	38.9 (20.7–57.1)	
Yes	20 (14.5)	19 (95.0)	13.4 (5.9–20.9)	
Chemotherapy				0.183
No	52 (37.7)	31 (59.6)	23.5 (14.5–32.5)	
Yes	86 (62.3)	45 (52.3)	37.8 (22.2–53.4)	

GC, gastric cancer; CI, confidence interval; pN, lymph node status; LNR, lymph node ratio; TNM staging, tumor-node-metastases staging; TRM staging, tumor-node-ratio-metastases staging; SAE, serious adverse event; OS, overall survival.

**Table II:** Independent prognostic factors of 138 GC patients identified by multivariate analysis.

Variables	χ^2^	Hazard ratio (95% CI)	P-value
TNM-based			
pN			0.004
N0 (reference)			
N1	0.356	1.342 (0.510–3.531)	0.551
N2	4.022	2.301 (1.019–5.193)	0.045
N3	11.120	3.319 (1.640–6.718)	0.001
Postoperative SAE			0.014
No (reference)			
Yes	6.034	1.991 (1.149–3.449)	
TRM-based			
LNR			<0.001
R0 (reference)			
R1	2.515	1.775 (0.873–3.609)	0.113
R2	18.771	5.636 (2.578–12.321)	<0.001
R3	34.116	15.113 (6.076–37.591)	<0.001
Distant metastases			0.006
No (reference)			
Yes	7.685	2.728 (1.342–5.548)	

GC, gastric cancer; CI, confidence interval; pN, lymph node status; SAE, serious adverse event; LNR, lymph node ratio.

**Table III:** Predictive value of the factors assessed in ROC analysis.

		95% CI			
				
Staging systems	AUC	Lower	Upper	Std. error	P-value
TRM staging system	0.769	0.692	0.845	0.039	<0.001
TNM staging system	0.745	0.662	0.827	0.042	<0.001
LNR classification	0.724	0.641	0.807	0.042	<0.001
pN classification	0.704	0.615	0.792	0.045	<0.001

ROC, receiver operating characteristic; AUC, area under the curve; CI, confidence interval; Std. error, standard error; TRM staging, tumor-node ratio-metastases staging; TNM staging, tumor-node-metastases staging; LNR, lymph node ratio; pN, lymph node status.

**Table IV:** Information on LNR from previous studies and the present study.

Authors (ref.)	No. of patients	No. of removed LNs (range)	Cutoff points of LNR	5-year survival rates of R0, R1, R2, R3 (%)
Kim *et al*(19)	529	6^a^(1–104)	0, 0.30, 0.60	71.7, 35.7, 16.3, 0
Asoglu *et al*(20)	264	27^b^(16–75)	0, 0.10, 0.25	86.9, 81.1, 47.1, 24.7
Xu *et al*(21)	177	20^a^(16–53)	0, 0.10, 0.25	84.3, 71.1, 45.1, 24.2
Lee *et al*(22)	342	28.9^b^(16–98)	0, 0.30, 0.60	Unknown
Huang *et al*(23)	634	23^a^(5–61)	0, 0.20, 0.50	83.3, 68.4, 40.7, 17.2
Feng *et al*(24)	109	38.34^b^	0, 0.10, 0.25	58.8, 43.8, 25.0, 10.4
Lemmens *et al*(25)	880	7^a^ (unknown)	0, 0.20, 0.30	58, 50, 18, 11
Wang *et al*(16)	1343	15^b^(3–72)	0, 0.30, 0.60	77.5, 64.3, 39.7, 22.3
Qiu *et al*(26)	730	16^a^ (0–72)	0, 0.30, 0.60	72.1, 65.6, 30.3, 13.0
Present study	138	21^a^(16–47)	0, 0.50, 0.80	69.7, 52.9, 20.8, 0

a Median number of removed LNs;

b mean number of removed LNs.

LNR, lymph node ratio; LN, lymph node.

## References

[1] Jemal A, Bray F, Center MM, Ferlay J, Ward E, Forman D (2011). Global cancer statistics. CA Cancer J Clin.

[2] Ferlay J, Shin HR, Bray F, Forman D, Mathers C, Parkin DM (2010). Estimates of worldwide burden of cancer in 2008: GLOBOCAN 2008. Int J Cancer.

[3] Yang L (2006). Incidence and mortality of gastric cancer in China. World J Gastroenterol.

[4] Hartgrink HH, Jansen EP, van Grieken NC, van de Velde CJ (2009). Gastric cancer. Lancet.

[5] Wang XN, Liang H (2010). Some problems in the surgical treatment of gastric cancer. Chin J Cancer.

[6] Viudez-Berral A, Miranda-Murua C, Arias-de-la-Vega F (2012). Current management of gastric cancer. Rev Esp Enferm Dig.

[7] Lee JH, Kim KM, Cheong JH, Noh SH (2012). Current management and future strategies of gastric cancer. Yonsei Med J.

[8] Wu HL, Tian Q, Peng CW, Liu SP, Li Y (2011). Multivariate survival and outcome analysis of 154 patients with gastric cancer at a single Chinese institution. Asian Pac J Cancer Prev.

[9] Landry CS, Brock G, Scoggins CR, McMasters KM, Martin RN (2009). A proposed staging system for gastric carcinoid tumors based on an analysis of 1,543 patients. Ann Surg Oncol.

[10] Cammerer G, Formentini A, Karletshofer M, Henne-Bruns D, Kornmann M (2012). Evaluation of important prognostic clinical and pathological factors in gastric cancer. Anticancer Res.

[11] Washington K (2010). 7th edition of the AJCC cancer staging manual: stomach. Ann Surg Oncol.

[12] Sun Z, Wang ZN, Zhu Z (2012). Evaluation of the seventh edition of American Joint Committee on Cancer TNM staging system for gastric cancer: results from a Chinese monoinstitutional study. Ann Surg Oncol.

[13] Chae S, Lee A, Lee JH (2011). The effectiveness of the new (7th) UICC N classification in the prognosis evaluation of gastric cancer patients: a comparative study between the 5th/6th and 7th UICC N classification. Gastric Cancer.

[14] Ahn HS, Lee HJ, Hahn S (2010). Evaluation of the seventh American Joint Committee on Cancer/International Union Against Cancer Classification of gastric adenocarcinoma in comparison with the sixth classification. Cancer.

[15] Wang W, Sun XW, Li CF (2011). Comparison of the 6th and 7th editions of the UICC TNM staging system for gastric cancer: results of a Chinese single-institution study of 1,503 patients. Ann Surg Oncol.

[16] Kim SS, Choi BY, Seo SI (2011). The comparison between 6th and 7th International Union Against Cancer/American Joint Committee on Cancer Classification for Survival Prognosis of Gastric Cancer. Korean J Gastroenterol.

[17] Wang W, Xu DZ, Li YF (2011). Tumor-ratio-metastasis staging system as an alternative to the 7th edition UICC TNM system in gastric cancer after D2 resection - results of a single-institution study of 1343 Chinese patients. Ann Oncol.

[18] Wang J, Dang P, Raut CP (2012). Comparison of a lymph node ratio-based staging system with the 7th AJCC system for gastric cancer: analysis of 18,043 patients from the SEER database. Ann Surg.

[19] Lee SR, Kim HO, Son BH, Shin JH, Yoo CH (2012). Prognostic significance of the metastatic lymph node ratio in patients with gastric cancer. World J Surg.

[20] Liu X, Cai H, Shi Y, Wang Y (2012). Prognostic factors in patients with node-negative gastric cancer: a single center experience from China. J Gastrointest Surg.

[21] Diaz de Liaño A, Yarnoz C, Aguilar R, Artieda C, Ortiz H (2008). Rationale for gastrectomy with D2 lymphadenectomy in the treatment of gastric cancer. Gastric Cancer.

[22] D’Annibale A, Pende V, Pernazza G (2011). Full robotic gastrectomy with extended (D2) lymphadenectomy for gastric cancer: surgical technique and preliminary results. J Surg Res.

[23] Kim CY, Yang DH (2009). Adjustment of N stages of gastric cancer by the ratio between the metastatic and examined lymph nodes. Ann Surg Oncol.

[24] Asoglu O, Karanlik H, Parlak M (2009). Metastatic lymph node ratio is an independent prognostic factor in gastric cancer. Hepatogastroenterology.

[25] Xu DZ, Geng QR, Long ZJ (2009). Positive lymph node ratio is an independent prognostic factor in gastric cancer after d2 resection regardless of the examined number of lymph nodes. Ann Surg Oncol.

[26] Lee SY, Hwang I, Park YS, Gardner J, Ro JY (2010). Metastatic lymph node ratio in advanced gastric carcinoma: A better prognostic factor than number of metastatic lymph nodes?. Int J Oncol.

[27] Huang CM, Lin JX, Zheng CH (2010). Prognostic impact of metastatic lymph node ratio on gastric cancer after curative distal gastrectomy. World J Gastroenterol.

[28] Feng J, Wu YF, Xu HM, Wang SB, Chen JQ (2011). Prognostic significance of the metastatic lymph node ratio in T3 gastric cancer patients undergoing total gastrectomy. Asian Pac J Cancer Prev.

[29] Lemmens V, Dassen AE, van der Wurff A, Coebergh J, Bosscha K (2011). Lymph node examination among patients with gastric cancer: variation between departments of pathology and prognostic impact of lymph node ratio. Eur J Surg Oncol.

[30] Qiu MZ, Qiu HJ, Wang ZQ (2012). The tumor-log odds of positive lymph nodes-metastasis staging system, a promising new staging system for gastric cancer after D2 resection in China. PLoS One.

[31] Peng CW, Wang LW, zeng WJ, Yang XJ, Li Y (2013). Evaluation of the staging system for gastric cancer. J Surg Oncol.

